# Biologic-assisted immunotherapy for pediatric food allergy: a systematic review

**DOI:** 10.3389/falgy.2026.1781735

**Published:** 2026-06-22

**Authors:** Anthony Mayer

**Affiliations:** Idaho College of Osteopathic Medicine, Idaho, United States

**Keywords:** biologics, dupilumab, food allergy, omalizumab, oral immunotherapy, pediatric allergy

## Abstract

**Background:**

Food allergies affect up to 8% of children and are a major cause of anaphylaxis, reduced quality of life, and healthcare use. Oral immunotherapy (OIT) can induce desensitization but is limited by adverse reactions, tolerability challenges, and variable long-term outcomes. Emerging biologic therapies- particularly omalizumab and dupilumab- may improve the safety and efficiency of desensitization when used alone or with immunotherapy.

**Objective:**

To systematically review evidence on biologic-assisted immunotherapy and alternative desensitization approaches in pediatric food allergy, focusing on efficacy, sustained unresponsiveness, safety, treatment efficiency, and clinical applicability.

**Methods:**

A systematic review following PRISMA 2020 guidelines was conducted using PubMed, with supplementary searches of Google Scholar and SciSpace (2015–2025), to identify interventional studies of biologic therapies, OIT, sublingual immunotherapy (SLIT), epicutaneous immunotherapy (EPIT), and combination approaches in children < 18 years with IgE-mediated food allergy. Studies were synthesized using a narrative qualitative approach. Desensitization was defined as increased tolerated dose during active therapy; reaction threshold as the dose provoking symptoms; sustained unresponsiveness (SU) as absence of symptoms after treatment cessation; and tolerance as durable long-term nonreactivity independent of therapy. Risk of bias for randomized trials was assessed using the Cochrane Risk of Bias 2 (RoB2) tool.

**Results:**

Of 505 records, 17 studies met inclusion criteria. Omalizumab-assisted OIT demonstrated higher desensitization rates (approximately 67%–85%) and fewer adverse events compared with OIT alone (approximately 30%–40%), though effect sizes varied. Dupilumab showed modest improvements in tolerability with smaller effects on desensitization. Omalizumab monotherapy increased reaction thresholds across multiple allergens. SLIT and EPIT demonstrated favorable safety with moderate efficacy, particularly in younger children.

**Conclusion:**

Evidence suggests biologic-assisted immunotherapy- particularly omalizumab with OIT- may improve desensitization and safety in pediatric food allergy, though findings are limited by heterogeneity, sample size, and risk of bias. Larger trials are needed to confirm long-term durability, optimize protocols, and assess cost-effectiveness. SLIT and EPIT may offer safer alternatives for selected high-risk populations.

## Introduction

Food allergies are an increasingly prevalent condition, affecting up to 8% of children in the United States and leading to a growing burden on patients and healthcare systems through increased emergency department visits and reduced patient and family quality of life (18). While avoidance and emergency preparedness remain the backbone of management, these approaches do not alter underlying immunologic sensitivity or disease progression. Over the last decade, the focus of pediatric allergy treatment has shifted from avoidance to active desensitization through immunotherapy, aiming to induce allergen tolerance and reduce anaphylaxis risk.

Oral immunotherapy (OIT) is currently the most established desensitization strategy. It works by gradually introducing small doses of the allergen to raise the threshold of reactivity in each patient. OIT has proven efficacy, but it is limited by high rates of reactions, including anaphylaxis and gastrointestinal distress, as well as lengthy treatment protocols, variable long-term results, and poor adherence. Many children discontinue OIT due to intolerable side effects or parental anxiety about dosing safety. This highlights the need for alternative immunotherapy approaches, particularly for children with severe or multiple food allergies.

In this context, biologic therapies have gained attention as adjuncts to OIT. By modulating immune pathways central to allergic inflammation, biologics may enhance desensitization efficacy while also mitigating adverse reactions associated with traditional OIT. Monoclonal antibodies- most notably omalizumab and, to a lesser extent, dupilumab- represent the most promising drug therapies for biologic-assisted immunotherapy in pediatric populations. Omalizumab is an anti-IgE monoclonal antibody that binds free IgE, preventing its interaction with the high-affinity Fc*ε*RI receptor on mast cells and basophils. This blockade decreases downstream allergic inflammation and reactivity. Initially approved for allergic asthma and chronic urticaria, omalizumab has emerged as a potential adjunct through evidence demonstrating facilitation of rapid and safer desensitization when used alongside OIT in children with peanut, milk, or multiple food allergies (10, 18). Beyond reducing reaction frequency, omalizumab can allow patients to reach maintenance doses faster and with fewer discontinuations.

Dupilumab, an IL-4 receptor *α* antagonist that inhibits IL-4 and IL-13 pathways, reduces Th2-mediated inflammation and IgE production. It is used clinically across several allergic diseases- including atopic dermatitis, asthma, and eosinophilic esophagitis- making it a candidate for food allergy treatment (3). Early data suggest dupilumab may improve OIT tolerability by reducing gastrointestinal and respiratory symptoms associated with immune activation, but evidence remains limited and its role as monotherapy or adjunct is still being defined.

Sublingual immunotherapy (SLIT) and epicutaneous immunotherapy (EPIT) deliver smaller allergen doses via oral mucosa or skin and generally achieve lower desensitization thresholds than OIT, while offering favorable safety profiles- attributes that may make them appropriate for younger or higher-risk children who cannot tolerate standard OIT protocols (5, 7).

Despite emerging data, consolidated comparisons between biologic-assisted therapies and traditional or alternative desensitization methods in pediatric populations remain sparse. This review synthesizes randomized controlled trials, interventional studies, and systematic reviews/meta-analyses to evaluate efficacy, safety, and clinical applicability of biologic-assisted and alternative immunotherapies in pediatric food allergy.

## Methods

### Study design and reporting framework

This systematic review was conducted in accordance with PRISMA 2020 guidelines. The review question was structured using PICOS criteria: pediatric patients with IgE-mediated food allergy (Population), biologic therapies and immunotherapy modalities (Intervention), standard care or alternative immunotherapy strategies (Comparator), outcomes including desensitization, sustained unresponsiveness, safety, and quality of life (Outcomes), across interventional study designs (Study design). The review question, eligibility criteria, and study design parameters were defined prior to formal screening based on the PICOS framework. Minor refinements were made during full-text review to improve methodological consistency and relevance; these adjustments were applied systematically across all studies rather than selectively.

### Search strategy

A structured literature search was conducted primarily using PubMed. Supplementary searches were performed using Google Scholar and SciSpace to identify additional relevant studies. Searches were restricted to studies published between 2015 and 2025. Google Scholar searches were used only as supplementary sources; Full reproducible search strings are provided in the Supplementary Methods. Searches were standardized within PubMed, and supplementary platforms were used only for additional study identification rather than primary reproducible search outputs.

### Eligibility criteria

Studies were included if they:

- Evaluated biologic and/or desensitization-based interventions
- **Included** pediatric participants (< 18 years) with diagnosed food allergies
- Reported measurable clinical outcomes (anaphylaxis risk, QoL, threshold of reactivity, SU, treatment-related adverse events)
- Were peer-reviewed publications in English between 2015 and 2025
- Used interventional study designs

Note: Studies including mixed-age populations were retained when pediatric participants were included or when findings were applicable to pediatric clinical interpretation.

Studies were excluded if they:

- Focused **exclusively** on adults (> 18 years)
- Were case reports/series with < 10 participants
- Were animal or *in vitro* studies
- Were non-interventional/descriptive only
- Were abstract-only or non–peer-reviewed
- Lacked a defined intervention or measurable clinical outcomes
- Were duplicate publications

### Study selection

**Figure 1:**
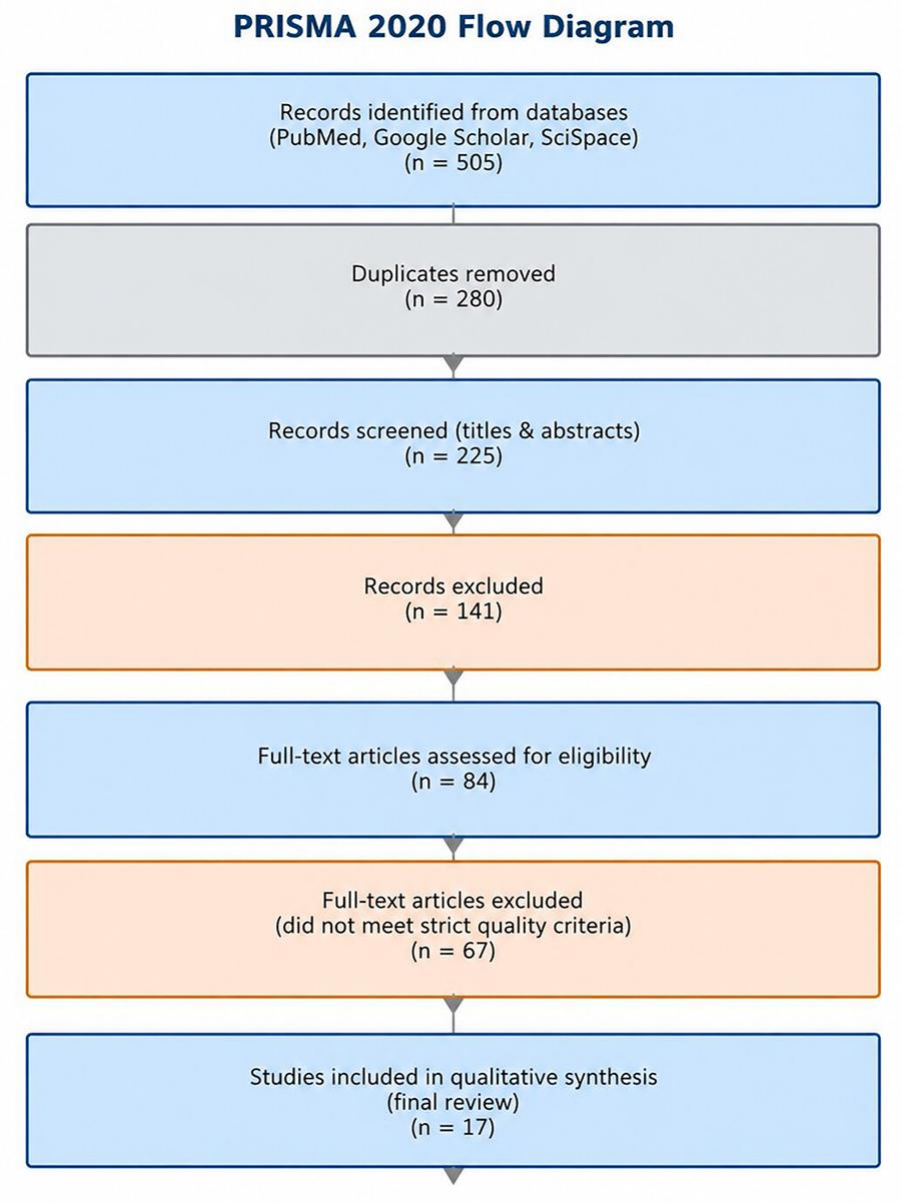
PRISMA 2020 flow diagram of study selection. Flow diagram showing study identification, screening, eligibility, and inclusion process (records identified *n* = 505; studies included *n* = 17).

Screening was performed in two stages (title/abstract followed by full-text review) using predefined eligibility criteria. Initial database searches yielded 505 records. After removal of 280 duplicates, 225 unique records were screened by title and abstract; 84 full texts were assessed and 67 were excluded for not meeting inclusion criteria, leaving 17 included studies. The study selection process is summarized in Figure 1 (PRISMA flow diagram). All screening and eligibility assessments were performed by a single author. No formal independent cross-checking of screening or data extraction was performed. The sample-size thresholds listed below were applied as quality filters during full-text assessment to prioritize methodologically robust trials rather than as an initial exclusion at title/abstract screening. These thresholds were applied consistently across all studies and were intended to prioritize interpretability rather than selectively exclude studies based on results.

- Sample size > 25 for RCTs, systematic reviews, or large cohort studies
- Minimum sample size > 20 for interventional studies and > 100 for observational studies
- Focus on biologics in combination with immunotherapy or as monotherapy
- Inclusion of primary outcomes such as SU, threshold changes, anaphylaxis rates, QoL, and relevant biomarkers

### Data extraction and synthesis

Data extracted from each included study comprised study design, population characteristics, intervention type, comparator (if applicable), primary and secondary outcomes, main findings, treatment duration, and relevant methodological details. Studies were categorized by design and intervention type. Given heterogeneity in study design, populations, allergens, outcome measures, and treatment protocols, a narrative qualitative synthesis approach was employed rather than quantitative meta-analysis.

### Risk of bias and quality assessment

We applied the Cochrane Risk of Bias 2 (RoB2) tool to all included randomized controlled trials.

We assessed risk of bias for randomized trials using the Cochrane Risk of Bias 2 (RoB2) tool across the five domains (randomization process; deviations from intended interventions; missing outcome data; measurement of outcomes; selection of the reported result). Because screening and extraction were single-author for this review, RoB2 criteria were applied systematically and domain judgments recorded. Nonrandomized and small interventional studies were appraised narratively, focusing on enrollment, outcome ascertainment, completeness of outcome data, and conflicts of interest. Summary judgments are presented in Table 1 (RoB2 summary) below.

**Table 1:** RoB2 summary (randomized trials). (Condensed per–trial domain judgments with brief rationale). Risk of bias (RoB2) assessment summary.

Study	Randomization process	Deviations from intended interventions	Missing outcome data	Measurement of outcomes	Selection of reported result	Overall RoB
Vickery et al. PALISADE (16)	Low risk — central computerized randomization with allocation concealment and prespecified stratification described.	Low risk — double-blind, placebo-controlled with protocolized dosing and blinded staff, minimizing deviations.	Low risk — ITT analyses prespecified; withdrawals and reasons reported; sensitivity analyses performed.	Low risk — DBPCFC assessed using standardized blinded procedures.	Low risk — outcomes and hierarchical testing prespecified and reported accordingly.	Low risk — large, well-conducted phase 3 RCT with transparent reporting; industry funding declared.
Wood et al. (18) (omalizumab monotherapy)	Low risk — central randomization with stratification and allocation methods described.	Low risk — double-blind design; COVID-related amendments prespecified and handled per protocol.	Low risk — attrition and handling of missing data prespecified and reported.	Low risk — DBPCFC primary outcomes with blinded assessors and standard protocols.	Low risk — primary and secondary outcomes reported per statistical analysis plan.	Low risk — robust multicenter trial; pandemic interruptions transparently managed.
MacGinnitie et al. (10)	Low risk — randomization and blinding described.	Some concerns — small sample and inclusion of open-label/extension elements may influence later comparisons.	Some concerns — withdrawals and protocol crossovers reduce precision despite reporting.	Low risk — DBPCFC outcomes objective and assessed blinded where applicable.	Low risk — primary endpoints prespecified; exploratory analyses included.	Some concerns — randomized and blinded for primary endpoints, but small sample and open-label elements limit certainty for maintenance outcomes.
Wood et al. (17) (milk OIT + omalizumab)	Low risk — randomized, double-blind, placebo-controlled design.	Some concerns — open-label extension/unblinding may bias maintenance comparisons.	Some concerns — moderate attrition for longer-term endpoints affects precision.	Low risk — DBPCFC outcomes assessed with blinding.	Low risk — outcomes align with protocol and supplementary tables provided.	Some concerns — valid randomized design, but maintenance-phase inference limited by unblinding and attrition.
Andorf et al. (1)	Low risk — block randomization (3:1) with allocation prepared by blinded biostatistician and described.	Low risk — double-blind design with no unblinding during trial.	Low risk — all randomized participants completed primary endpoint assessment; ITT applied.	Low risk — DBPCFC primary outcomes with blinded assessors and standardized dosing.	Low risk — primary and key secondary endpoints prespecified and reported with statistical detail.	Low risk — randomized, double-blind phase 2 trial with complete follow-up; modest sample but transparent reporting.
Chinthrajah et al. (3)	Low risk — randomized with stratification and allocation concealment described.	Some concerns — complex design (re-randomization, pandemic-related mFAS adjustments) may introduce deviations.	Some concerns — imputation and analytic complexity reduce clarity for some endpoints.	Low risk — DBPCFC outcomes assessed with blinding.	Some concerns — multiple subgroup analyses increase risk of selective reporting.	Some concerns — strong randomized design, but analytic complexity warrants cautious interpretation.
Sindher et al. (14)	Low risk — randomized components described.	Some concerns — pragmatic “real-world” heterogeneity increases potential deviations.	Some concerns — small sample size and variable follow-up reduce precision.	Low risk — objective clinical thresholds used.	Some concerns — exploratory QoL/immunologic endpoints increase multiplicity risk.	Some concerns — improved external validity but reduced internal control.
Frischmeyer-Guerrerio et al. (4)	Low risk — randomization and allocation concealment described.	Some concerns — mechanistic focus and exploratory elements may introduce deviations.	Some concerns — modest sample size limits precision.	Low risk — objective biomarker and DBPCFC measures used.	Some concerns — multiple exploratory biomarker analyses increase selective reporting risk.	Some concerns — mechanistic strengths but small sample and exploratory analyses limit certainty.

DBPCFC, double-blind placebo-controlled food challenge; ITT, intention-to-treat; mFAS, modified full analysis set.

Certain small randomized trials (e.g., rapid desensitization-MacGinnitie and related Sindher pilot studies) are grouped due to similar design characteristics, limited sample sizes, and comparable methodological features. Nonrandomized/interventional studies (e.g., Sindher (14) phase-2 interventional real-world study; Hemler (5) case series) were appraised narratively and are not included in the RoB2 table. Martorell—Calatayud 2016 full text was not available to the reviewer and is noted as excluded from detailed RoB2 extraction (refer to Limitations/Table footnotes).

### Transparency and protocol registration

AI-assisted tools were used only for early topic exploration and reference organization; all study selection, data extraction, synthesis, interpretation, and final writing were performed manually by the author. Although this review was not prospectively registered in PROSPERO, the review question, eligibility criteria, and search strategy were defined prior to formal screening; registration was not completed due to timing constraints and is acknowledged as a limitation.

### Limitations of methodology

This review has several limitations. First, heterogeneity among included studies (sample size, allergen type, dosing regimens, outcome definitions) limited synthesis and precluded meta-analysis. Many trials were of moderate duration (16–52 weeks), restricting conclusions regarding long-term sustained unresponsiveness. Second, study selection and data extraction were performed by a single author, which introduces potential selection bias and limits reproducibility; duplicate independent screening/extraction was not performed. Although RoB2 was applied to RCTs (Table 1), the single-author workflow remains a limitation. Third, this review was not prospectively registered in PROSPERO; predefined inclusion criteria and structured screening were used to mitigate selective reporting. Publication bias may favor positive studies. Finally, Martorell—Calatayud 2016 full text was unavailable and therefore excluded from detailed extraction and RoB2; this is noted in Table 3 footnotes.

## Results

A total of 17 studies, including randomized controlled trials, interventional studies, and systematic reviews/meta-analyses, were included in the final qualitative synthesis. Study characteristics are summarized in Table 2, with key outcomes presented in Table 3. Study designs ranged from randomized controlled trials and prospective case series to meta-analyses and systematic reviews, focusing primarily on omalizumab and dupilumab as adjuncts or monotherapies for food allergy management. Results are presented thematically according to therapeutic approach.

**Table 2:** Characteristics of included meta-analyses and systematic reviews.

Author(s) & Year	Study Type	Intervention/Focus	Population (n, age range)	Primary Outcomes	Main Findings (Condensed)
Buono et al. 2025 (2)	Meta-analysis & systematic review	Omalizumab + OIT	436 participants, mixed-age population including pediatric subgroups	Sustained unresponsiveness; Anaphylaxis risk	Omalizumab + OIT improved desensitization thresholds and reduced adverse events. Peanut allergy patients benefited most; reactions were mild and manageable.
Mustafa et al. 2025 (11)	Meta-analysis	Omalizumab as adjunct to OIT	323 across 9 studies (pediatric focus)	Desensitization rate; safety profile	Combination therapy was associated with improved OIT efficacy and reduced adverse events; heterogeneity limits generalizability of findings.
Riggioni et al. 2024 (13)	Systematic review/Meta-analysis	Biologics and Allergen-specific Immunotherapy (OIT, EPIT, SLIT)	Variable (mixed pediatric & adult)	Desensitization; Sustained unresponsiveness; Quality of Life	Both biologics and OIT were effective during active treatment, but long-term tolerance and protocol standardization remain unresolved.
Nurmatov et al. 2025 (12)	Meta-analysis	Omalizumab monotherapy and as adjunct	1010 participants (all ages)	Desensitization and tolerance	Omalizumab improves desensitization and tolerance while maintaining a favorable safety profile; cost-effectiveness data are lacking.

**Table 3:** Characteristics and outcomes of randomized and interventional studies.

Study	Design	Population	Primary outcome	Secondary outcomes	Safety
MacGinnitie et al. (10) (omalizumab + peanut OIT)	RCT (double-blind, placebo-controlled)	Total *n* = 37 (omalizumab *n* = 29; placebo *n* = 8)	Tolerated 2000mg peanut 6 weeks after stopping study drug — omalizumab 23/29 (79.3%) vs. placebo 1/8 (12.5%); *p* < 0.01 (ITT).	Passed 4000 mg OFC — 22/29 (75.9%) vs. 1/8 (12.5%); *p* = 0.002. Median tolerated dose on rapid desensitization day: 250 mg vs. 22.5 mg; *p* = 0.00027.	Reaction-per-dose rate 7.8% vs. 16.8% (OR 0.57, *p* = 0.15); 14 epinephrine reactions in 8 subjects; 3 participants developed persistent GI symptoms consistent with EoE.
Wood et al. (18) (omalizumab monotherapy)	Phase 3 multicenter RCT	Pediatric cohort *n* = 177	Tolerated single 600 mg peanut at end of treatment — omalizumab 67% vs. placebo 7%; *p* < 0.001.	—	Injection-site reactions more common in omalizumab arm; serious adverse events uncommon; no new safety signals reported.
Wood et al. (17) (omalizumab + milk OIT)	RCT (double-blind)	Total *n* = 57 (omalizumab *n* = 28; 27 initiated dosing; placebo *n* = 29)	Month 28 (10 g OFC) passed — omalizumab 24/27 (88.9%) vs. placebo 20/28 (71.4%); *p* = 0.18.	Month 32 sustained unresponsiveness — omalizumab 13/27 (48.1%) vs. placebo 10/28 (35.7%); *p* = 0.42. Faster time to maintenance (median doses 198 vs. 225; *p* = 0.008).	Escalation symptomatic doses 8.5% vs. 26.1%; epinephrine-requiring doses: 2 doses in 2 omalizumab subjects vs. 18 doses in 9 placebo subjects.
Andorf et al. (1) (omalizumab + multifood OIT)	Phase 2 RCT	Total *n* = 48 (omalizumab *n* = 36; placebo *n* = 12)	Passed DBPCFC to ≥ 2 foods (2 g each) — omalizumab 30/36 (83%) vs. placebo 4/12 (33%); OR 10.0 (95% CI 1.8–58.3); *p* = 0.004.	Passed 4 g for ≥ 2 foods — 30/36 vs. 4/12; *p* = 0.004. Time to maintenance HR 5.36 (95% CI 1.8–15.99); *p* = 0.001.	No grade ≥ 3 adverse events or serious adverse events reported; lower rates of gastrointestinal and respiratory adverse events in the omalizumab group.
Chinthrajah et al. (3) (dupilumab + OIT)	Multicenter RCT	Total *n* = 148 (6–17 years)	Up-dosing endpoint (2044mg) — approximately 20% higher DBPCFC pass rate with dupilumab + OIT vs. placebo + OIT; *p* < 0.05.	Maintenance effects smaller and not statistically significant.	Anaphylaxis rates similar between groups; adverse event profile consistent with dupilumab.
Vickery et al. PALISADE (AR101) (16)	Phase 3 RCT	Total *n* = 551; children 4–17 *n* = 496	Tolerated single 600 mg DBPCFC at exit — AR101 67.2% (333/496) vs. placebo 4.0% (10/250); *p* < 0.001.	—	Typical OIT adverse event profile; gastrointestinal events common; epinephrine used in some participants; EoE rare.
Sindher et al. (14) (multi-OIT + omalizumab)	Phase 2 randomized pragmatic study	Total *n* ≈ 60 (mixed age population)	Protocol-defined desensitization and feasibility outcomes demonstrated improved tolerability and earlier dose progression compared with control approaches.	—	Improved escalation tolerability; fewer moderate reactions compared with historical cohorts.
Hemler et al. (5) (early-age peanut OIT)	Prospective interventional case series	Total *n* = 27 (infants/toddlers)	Maintenance achievement — 90% reached maintenance; OFC subset: 8/8 tolerated 6000 mg.	—	Mostly mild adverse events; no cases of EoE reported.
Kim et al. (7) (peanut SLIT)	Randomized, placebo-controlled trial	Total *n* = 50 (1–4 years)	Cumulative tolerated dose — SLIT median 4443 mg vs. placebo 143 mg; desensitization 60% vs. 0%.	—	Favorable safety profile; no severe reactions reported.
MacGinnitie rapid desensitization pilot trials	Pilot randomized feasibility trials	Small samples (∼20–40 participants per study)	Facilitated rapid desensitization with higher tolerated doses during escalation phases.	—	Reduced escalation reactions with omalizumab adjunct; small sample sizes limit inference regarding serious adverse events.
Martorell-Calatayud et al. 2016 (omalizumab-assisted OIT)	Multicenter RCT	Total *n* ≈ 40	Reported summary suggests facilitated desensitization.	—	Safety profile appears favorable in available summaries; detailed outcomes could not be independently verified due to unavailable full text.

### Theme 1: omalizumab + OIT

Across multiple included trials, combining omalizumab with OIT was associated with higher desensitization rates and improved safety metrics compared with OIT alone; however, effect sizes and outcomes varied by trial design, population, allergens, and dosing protocols (see Table 3 and RoB2 Table 1). For example, Andorf et al. (1) reported that a greater proportion of participants in the omalizumab group (83%) passed DBPCFC to at least two allergens compared with placebo (33%). Additional contextual literature not included in the PRISMA synthesis is summarized in Table 4.

**Table 4:** Contextual/Non-PRISMA literature.

Author(s) & Year	Article Type	Key Clinical Argument/Relevance
Vickery et al. 2024 (15)	Rostrum (Expert Perspective)	Summarized the OUTMATCH trial design and highlighted omalizumab's emerging role as a bridge to safer, broader desensitization therapies.
Kulis et al. 2025 (8)	Rostrum (Comparative Perspective)	Compared anti-IgE therapy and AIT modalities; discussed combination strategies and future immunomodulatory targets.
Labrosse et al. 2017 (9)	Review (Expert Summary)	Evaluated omalizumab's impact on OIT safety and efficacy; emphasized quality-of-life improvements and need for standardized protocols.
Indolfi et al. 2025 (6)	Narrative Review with Empirical Data	Highlighted Omalizumab's efficacy in enhancing AIT (Allergen Immunotherapy) safety and outcomes; advocated individualized protocol design for children.

Table 1: RoB2 summary (inserted above).

### Theme 2: dupilumab + OIT

Dupilumab targets IL-4/IL-13 and may attenuate Th2 inflammation, potentially improving OIT tolerability. Chinthrajah et al. (3) reported an approximately 20% absolute increase in the proportion tolerating 2044mg peanut protein during the up-dosing endpoint with dupilumab + OIT vs. OIT alone (*p* < .05). This result pertains to the primary up-dosing endpoint and should be interpreted with caution given trial complexity (re-randomization and maintenance subgroup analyses) and RoB2 domain considerations (Table 1).

### Theme 3: monotherapy biologics (omalizumab alone)

Several trials evaluated omalizumab monotherapy, primarily assessing its ability to increase reaction thresholds during active treatment. In a multicenter phase 3 trial ((18); *n* = 177 pediatric participants), a 16-week omalizumab regimen significantly increased reaction thresholds for multiple foods vs. placebo (e.g., 67% tolerated ≥ 600 mg peanut vs. 7% placebo). Importantly, the study design primarily evaluates the ability of omalizumab to transiently increase tolerance to allergen exposure during active treatment rather than induction of sustained immunologic tolerance. Allergen challenge was performed at the end of the treatment period, in close temporal proximity to the final omalizumab doses, and therefore reflects pharmacologic suppression of allergic reactivity rather than durable desensitization. The duration of post-treatment refractoriness to allergen challenge was not the primary focus of this trial and remains uncertain.

These monotherapy trials were judged as low risk or having some concerns based on RoB2 criteria (Table 1).

### Theme 4: alternative immunotherapies (SLIT, EPIT, OIT alone)

SLIT and EPIT offer additional options. Kim et al. (7) reported higher cumulative tolerated doses in peanut SLIT participants vs. placebo in young children. Hemler et al. (5) reported high maintenance rates with early-age peanut OIT in a prospective case series. These approaches generally show favorable safety profiles but lower potency compared with OIT.

### Overall summary

The evidence indicates that biologic-assisted immunotherapy- particularly omalizumab adjunctive therapy- suggests generally consistent signals of improved desensitization and safety across studies, though with variability of magnitude. Dupilumab shows potential for tolerability improvements but with smaller magnitude effects on desensitization.

## Discussion

### Summary of main findings

This review synthesized evidence from 17 studies examining biologic-assisted and alternative immunotherapy strategies. Several randomized control trials (e.g., PALISADE, Wood (18)) support omalizumab's ability to increase reaction thresholds and to facilitate safer OIT escalation; however, smaller pilot trials and real-world series contribute heterogeneity. Importantly, the study design primarily evaluates the ability of omalizumab to transiently increase tolerance to allergen exposure during active treatment rather than induction of sustained immunologic tolerance. Allergen challenge was performed at the end of the treatment period, in close temporal proximity to the final omalizumab doses, and therefore reflects pharmacologic suppression of allergic reactivity rather than durable desensitization. The duration of post-treatment refractoriness to allergen challenge was not the primary focus of this trial and remains uncertain. Overall conclusions reflect promising efficacy signals tempered by limitations in generalizability and long-term durability.

### Clinical and scientific implications

Omalizumab adjunct therapy enhances safety and speed of OIT escalation by suppressing IgE-mediated effector cell activation, potentially enabling more patients (including those with severe or multiple food allergies) to attempt desensitization. Dupilumab's IL-4/IL-13 blockade offers a complementary mechanism that may reduce OIT-related GI and respiratory symptoms and may be useful in patients with comorbid atopic disease.

### Comparison with prior literature

Recent meta-analyses and systematic reviews similarly report that omalizumab improves desensitization outcomes and reduces adverse events (2, 11–13, 19), while evidence for dupilumab remains emergent. Alternative immunotherapies show consistent safety advantages but lower potency compared with OIT. Importantly, most included trials evaluate desensitization and reaction thresholds under controlled conditions rather than real-world dietary incorporation of allergenic foods. In clinical practice, patient goals often extend beyond threshold increases to sustained integration of foods into the diet, which is not consistently captured in trial endpoints and may limit generalizability.

### Mechanistic and theoretical insights

Omalizumab neutralizes circulating IgE and downregulates Fc*ε*RI expression on mast cells and basophils, reducing effector responses and facilitating safer allergen exposure during OIT. Dupilumab modulates upstream Th2 signaling, decreasing class switching and type-2 inflammation-rationales that support combined or sequential biologic strategies.

### Limitations

Limitations include study heterogeneity, moderate trial durations limiting long-term inferences, single-author screening/extraction, potential publication bias, and unavailability of some full texts (e.g., Martorell—Calatayud 2016), which was excluded from detailed extraction. This review was conducted within the constraints of a single-author systematic framework. While methodological rigor was strengthened through predefined eligibility criteria, structured screening, and application of the RoB2 tool for randomized trials, the absence of duplicate independent review limits reproducibility compared with multi-reviewer systematic reviews.

### Future directions

Future research should include large, randomized comparative trials of biologic-assisted OIT, biologic monotherapy, and alternative immunotherapies, with standardized outcome definitions (desensitization thresholds, sustained unresponsiveness), longer follow-up, and cost-effectiveness analyses. Additionally, further work is needed to optimize dupilumab therapy, including evaluation of combination strategies with OIT, sequencing relative to allergen exposure, and identification of patient subgroups most likely to benefit based on underlying Th2 inflammatory profiles. Refinement of patient selection is also critical. Restricting biologic therapy to highly allergic or multi-food allergic populations may enhance observed efficacy and safety signals in clinical trials, but may limit generalizability to broader pediatric populations. Stratified trial designs incorporating baseline IgE levels, clinical severity, and multi-food allergy status may help balance internal validity with real-world applicability.

## Conclusion

Biologic-assisted immunotherapy, especially omalizumab as an adjunct to OIT, shows promising improvements in desensitization rates and safety in pediatric food allergy trials. However, heterogeneity, sample size limitations, and methodological constraints temper certainty. Larger, standardized, long-term trials are needed to define sustained unresponsiveness durability, optimal protocols, comparative effectiveness, and cost-effectiveness before broad clinical implementation.

## Data Availability

Publicly available datasets were analyzed in this study. This data can be found here: Not applicable. All data analyzed in this study are derived from publicly available, previously published articles cited in the reference list. No original datasets were generated or deposited in a repository.

## References

[1] Andorf S Purington N Block W Long AJ Tupa D Brittain E. Anti-IgE treatment with oral immunotherapy in multifood allergic participants: a double-blind, randomised, controlled trial. Lancet Gastroenterol Hepatol. (2018) 3:85. 10.1016/S2468-1253(17)30392-8 29242014 PMC6944204

[2] Buono EV Giannì G Scavone S Esposito S Caffarelli C. Omalizumab and oral immunotherapy in IgE-mediated food allergy in children: a systematic review and a meta-analysis. Pharmaceuticals. (2025) 18:437. 10.3390/ph18030437 40143213 PMC11946088

[3] Chinthrajah RS Sindher SB Nadeau KC Leflein JG Spergel JM Petroni D. Dupilumab as an adjunct to oral immunotherapy in pediatric patients with peanut allergy. Allergy. (2024) 80:827. 10.1111/all.16420 39673367 PMC11891407

[4] Frischmeyer-Guerrerio PA Masilamani M Gu W Brittain E Wood R Kim J. Mechanistic correlates of clinical responses to omalizumab in the setting of oral immunotherapy for milk allergy. J Allergy Clin Immunol. (2017) 140(4):1043–1053.e8. 10.1016/j.jaci.2017.03.028 28414061 PMC5632581

[5] Hemler J Minnicozzi S Carey A Braden K Boyd K. Early age peanut oral immunotherapy is safe and effective at achieving desensitization in 27 pediatric patients with peanut allergy. Pediatr Allergy Immunol. (2024) 35:e14273. 10.1111/pai.14273 39487696

[6] Indolfi C Perrotta A Dinardo G Klain A Grella C Palumbo P. Omalizumab in food allergy in children: current evidence and future perspectives. Life. (2025) 15(5):681. 10.3390/Life15050681 40430110 PMC12113005

[7] Kim EH Bird JA Keet CA Virkud YV Herlihy L Ye P. Desensitization and remission after peanut sublingual immunotherapy in 1- to 4-year-old peanut-allergic children: a randomized, placebo-controlled trial. J Allergy Clin Immunol. (2024) 153(1):173–181.e10. 10.1016/j.jaci.2023.08.032 37815782 PMC10872748

[8] Kulis MD Humphrey JR Krempski JW Kim EH Smeekens JM. Anti-IgE therapy versus allergen-specific immunotherapy for food allergy: weighing the pros and cons. Front Immunol. (2025) 16:1617153. 10.3389/fimmu.2025.1617153 40766311 PMC12321543

[9] Labrosse R Graham F Des Roches A Bégin P. The use of omalizumab in food oral immunotherapy. Arch Immunol Ther Exp. (2017) 65(3):189–99. 10.1007/s00005-016-0420-z 27628022

[10] MacGinnitie AJ Rachid R Gragg H Little SV Lakin P Cianferoni A. Omalizumab facilitates rapid oral desensitization for peanut allergy. J Allergy Clin Immunol. (2017) 139(3):873–881.e8. 10.1016/j.jaci.2016.08.010 27609658 PMC5369605

[11] Mustafa AAK Elagab EA Elhagaly AMA Bilal AAMA Hassan DHM Kamel AAA. The role of omalizumab as an adjunct to oral immunotherapy in pediatric food allergy: a systematic review. Cureus. (2025) 17:e85550. 10.7759/cureus.85550 40630369 PMC12237216

[12] Nurmatov UB Lo Scalzo L Galletta F Krasnenkova M Arasi S Ansotegui IJ. Biologics in IgE-mediated food allergy: a systematic review and meta-analysis of interventional studies. World Allergy Organ J. (2025) 18(7):101069. 10.1016/j.waojou.2025.101069 40510735 PMC12158532

[13] Riggioni C Otón T Carmona L Toit GD Skypala I Santos AF. Immunotherapy and biologics in the management of IgE-mediated food allergy: systematic review and meta-analyses of efficacy and safety. Allergy. (2024) 79:2097. 10.1111/all.16129 38747333

[14] Sindher SB Kumar D Cao S Purington N Long A Sampath V. Phase 2, randomized multi oral immunotherapy with omalizumab “real life” study. Allergy. (2022) 77(6):1873–84. 10.1111/all.15217 35014049

[15] Vickery BP Bird JA Chinthrajah RS Jones SM Keet C Kim E. Omalizumab implementation in practice: lessons learned from the OUtMATCH study. J Allergy Clin Immunol Pract. (2024) 12:2947. 10.1016/j.jaip.2024.08.056 39293782 PMC11560495

[16] Vickery BP Vereda A Casale TB Beyer K Toit GD Hourihane JO. AR101 oral immunotherapy for peanut allergy. N Engl J Med. (2018) 379:1991. 10.1056/NEJMOA1812856 30449234

[17] Wood RA Kim JS Lindblad R Nadeau K Henning AK Dawson P. A randomized, double-blind, placebo-controlled study of omalizumab combined with oral immunotherapy for the treatment of cow’s milk allergy. J Allergy Clin Immunol. (2016) 137(4):1103–1110.e11. 10.1016/j.jaci.2015.10.005 26581915 PMC5395304

[18] Wood RA Togias A Sicherer SH Shreffler WG Kim EH Jones SM. Omalizumab for the treatment of multiple food allergies. N Engl J Med. (2024) 390(10):889–99. 10.1056/NEJMoa2312382 38407394 PMC11193494

[19] Zuberbier T Wood RA Bindslev-Jensen C Fiocchi A Chinthrajah RS Worm M. Omalizumab in IgE-mediated food allergy: a systematic review and meta-analysis. J Allergy Clin Immunol Pract. (2023) 11(4):1134–46. 10.1016/j.jaip.2022.11.036 36529441

