# Supplementary material for: Biologic-assisted immunotherapy for pediatric food allergy: a systematic review
Source: Front Allergy. 2026 Jun 22;7:1781735. doi: 10.3389/falgy.2026.1781735 (PMC13333604; doi:10.3389/falgy.2026.1781735)
Supplement: Supplementary file 1 [file Table1.docx]

**Supplementary Methods**

## **Search Strategy**

A structured literature search was conducted using **PubMed as the primary biomedical database**, consistent with the approach described in the main Methods section. Supplementary searches were performed using Google Scholar and SciSpace to identify additional relevant studies and ensure comprehensive coverage.

Searches were restricted to studies published between **January 1, 2015 and December 31, 2025**, involving pediatric populations (<18 years) with IgE-mediated food allergy. Only English-language, human studies were considered.

## **PubMed Search Strategy (Primary, Reproducible)**

The following Boolean search strategy was applied in PubMed:

(“Food Hypersensitivity”[MeSH] OR “food allergy” OR “IgE-mediated food allergy”)
AND (“oral immunotherapy” OR “OIT” OR “sublingual immunotherapy” OR “SLIT” OR “epicutaneous immunotherapy” OR “EPIT”)
AND (“omalizumab” OR “dupilumab” OR “biologic therapy” OR “monoclonal antibody” OR “anti-IgE” OR “IL-4 receptor antagonist”)
AND (“child” OR “pediatric” OR “adolescent”)

Filters applied:

- English language
- Human studies
- Publication dates: 2015–2025

This strategy was designed to capture studies evaluating biologic therapies and immunotherapy modalities in pediatric food allergy populations.

## **Supplementary Search Strategies**

### **Google Scholar (Supplementary Source)**

Google Scholar was used to identify additional relevant studies not captured in database searches, including recently published articles and citation-linked records.

Representative search terms included combinations of:

- “oral immunotherapy,” “omalizumab,” “dupilumab”
- “pediatric food allergy,” “desensitization,” “anaphylaxis,” “reaction threshold,” “quality of life”

Search results were screened manually for relevance based on predefined eligibility criteria. Google Scholar was not used as a primary reproducible database but as a supplementary tool to enhance completeness of study identification.

### **SciSpace (Exploratory Supplementary Tool)**

SciSpace was used as an exploratory platform to identify potentially relevant publications and citation linkages. It was not used as a primary source for systematic retrieval but to supplement PubMed-based searches.

## **Study Identification Summary**

A total of **505 records** were identified across all sources. After removal of duplicates, **225 unique records** remained and were screened by title and abstract, followed by full-text review as described in the main Methods section and illustrated in the PRISMA flow diagram.
